# Mystery Behind Barrett's Esophagus: The Origin and Malignant Transformation of Esophageal Adenocarcinoma

**DOI:** 10.1055/s-0042-1758764

**Published:** 2022-12-21

**Authors:** Xiayao Diao

**Affiliations:** 1 Department of Thoracic Surgery, Peking Union Medical College Hospital, Chinese Academy of Medical Sciences and Peking Union Medical College, Beijing 100730, People's Republic of China

Esophageal cancer (EC) is the eighth most common cancer in the world, with an estimated 604,100 new cases in 2020, accounting for 3.1% of all cancer cases. 1 Esophageal squamous cell carcinoma (ESCC) and esophageal adenocarcinoma (EAC) are the two main histologic subtypes of EC. ESCC predominantly affects developing countries and accounts for more than 88.8% in Chinese EC patients, 2 3 while EAC predominantly affects developed countries and accounts for 80.1% in EC patients from United States. 4 5 Multiple risk factors, such as Barrett's esophagus, are associated with EC development. Barrett's esophagus is a typical metaplastic disease that begins at the gastroesophageal junctions with proximal displacement of the squamocolumnar junctions. Intestinal metaplasia increases the propensity for ECs, especially EACs, and may result from transcriptional switches within gastric cell types or products of intestinal cell types, but the exact origin is unclear. However, half of EAC patients were not observed to have Barrett's esophagus at the time of diagnosis. 6 7 Therefore, we cannot help but ask the following question: does Barrett's esophagus increase the risk of EAC? This question can be answered by determining the origin of Barrett's esophagus. Most scientists believe that Barrett's esophagus originates from many sources, such as various specific cell populations in the gastroesophageal junctions and esophageal submucosal glands. Lineage tracing studies in mouse models is the primary method for exploring Barrett's esophagus origin. However, the squamous pregastric keratinization and lack of esophageal submucosal glands make this animal model unable to fully mimic human gastroesophageal physiology. Additionally, isolation of esophageal submucosal glands from fresh human tissue is particularly difficult. All of these have become the major obstacles to lineage tracing studies.

In a study recently published in Science, titled “Molecular phenotyping reveals the identity of Barrett's esophagus and its malignant transition,” Nowicki-Osuch et al 8 successfully harvested the tissue samples across the gastroesophageal junction and isolated esophageal submucosal glands from patients and healthy individuals to explore the exact source of Barrett's esophagus. These tissue samples were analyzed by single-cell transcriptomic profiling, in silico lineage tracing of methylation, and somatic mutation/open chromatin array. The functional validation was performed in organoid models.

In brief, the authors immuno-stained pan-epithelial tissues, squamous tissues, columnar tissues, and esophageal submucosal glands of fresh human esophagus tissue with cadherin 1 (CDH1), keratin 5 (KRT5), keratin 8 (KRT8), and keratin 7 (KRT7) antibodies, respectively, and then used the three-dimensional confocal microscopy to identify and isolate the ductal cells, oncocytes, mucous cells, and myoepithelial cells. They observed a population of P63 ^+^ KRT5 ^+^ KRT7 ^+^ cells (transitional basal progenitor) in the intercalated and main duct of esophageal submucosal glands, which is consistent with previous studies and supports that this cell population contributes to Barrett's esophagus development. 9 However, they also observed that, contrary to previous studies, oncocytes (a population of cells characterized by centrally located nuclei and eosinophilic cytoplasm) were prevalent in Barrett's esophagus-free donors and often formed acini, indicated that they were associated with Barrett's esophagus development. Subsequent single cell-RNA sequencing identified four major cellular components of fresh dissociated esophageal submucosal glands that were quiescent (the vast majority of cells did not express the division marker MKI67) and expressed transcripts previously unrelated to esophageal submucosal gland, including AGR2, MUC5B, and KRT23. Furthermore, the authors discovered a distinct KRT7 ^high^ population (consisted of P63 ^+^ KRT5 ^+^ KRT7 ^+^ transitional basal cells, KRT7 ^+^ MUC4 ^+^ residual embryonic cells, and MUC5B ^high^ cells) at the normal squamocolumnar junctions of Barrett's esophagus-free donors. This subpopulation exhibited high similarity to the cells in esophageal submucosal gland, suggesting that it might have the same origin as esophageal submucosal glands. In contrast, they did not observe KRT7 ^high^ populations or any intermediate cell populations in the squamocolumnar junctions of patients with Barrett's esophagus, indicating that it is unlikely to transdifferentiate from normal esophagus cells to Barrett's esophagus cells. Surprisingly, the transcriptional, methylation, accessible chromatin, and clonal mutation profiles of Barrett's esophagus cells were remarkably similar to their normal gastric cardia counterparts. To further investigate the molecular mechanisms underlying Barrett's esophagus development, the authors performed gene set enrichment analysis and causal analysis of genes differentially expressed between different stages of Barrett's esophagus and normal gastric cardia, and the findings were elucidated in organoids established from normal gastric cardia tissues. They demonstrated that the exogenous expression of c-MYC and HNF4A in normal gastric cells drive the expression of Barrett's esophagus phenotype-associated genes. As EACs with Barrett's esophagus and Barrett's esophagus-free EACs have different clinical features and prognosis, whether all EACs were derived from Barrett's esophagus cells were further explored by performing multisubject single cell deconvolution analysis. These data suggested that EACs may originate from undifferentiated Barrett's esophagus cells, regardless of whether apparent Barrett's esophagus metaplasia is observed in diagnostic or pathological specimens.

Over the years, there have been only two hypotheses about the origin of Barrett's esophagus: residual embryonic cell hypothesis 10 and transitional basal cell hypothesis. 9 Due to the limitations in animal models and limited human samples, human esophageal submucosal glands have never been considered as the origin for Barrett's esophagus. KRT7-positive (KRT7 ^+^ ) cells in mouse gastroesophageal region have long been considered as a hallmark of Barrett's esophagus development. However, this study showed that KRT7 ^+^ cells are not only present in Barrett's esophagus, but also in squamocolumnar junctions and esophageal submucosal glands without Barrett's esophagus. Recently, a single Barrett's esophagus gland with the same mutational spectrum as an adjacent esophageal submucosal gland duct was identified in human tissue sections by a group from Cancer Research United Kingdom. 11 In addition, another group from University of Oxford 12 inferred that Barrett's esophagus may be derived from esophageal submucosal gland based on single cell-RNA sequencing data of human endoscopic pinch biopsies which included normal esophagus, normal gastric cardia, and Barrett's esophagus tissues. But none of these studies specifically isolated esophageal submucosal glands.

Although the expression of c-MYC and HNF4A in Barrett's esophagus has been reported, 13 14 they have always been regarded as the biomarkers of normal esophageal-derived cells. Unlike the studies mentioned above, this study used normal gastric cardia as a control tissue, which can more accurately reveal the origin of Barrett's esophagus. Furthermore, many previous studies have tried to explore the origin of EAC by analyzing Barrett's esophagus and EAC bulk tissues through gene expression microarray, 13 14 but they all failed. The emergence of single-cell profiling techniques provides strong technical support for solving this issue. This study adopted single-cell profiling technique and showed that even though the prognosis and evolutionary trajectories differed between EAC patients, their EACs were all derived from gastric cells through a Barrett's esophagus-like metaplasia. Moreover, this study also suggested that Barrett's esophagus may be an inevitable stage of tumor formation. This finding is consistent with The Cancer Genome Atlas study which concluded that EAC belongs to gastroesophageal adenocarcinoma spectrum. 15

The strengths of this study include: (1) human cells being analyzed were successfully isolated from superficial to submucosal compartments across the gastroesophageal junctions; (2) comprehensive multi-omic profiling was performed to reveal the origin of EAC. However, this study has several limitations: (1) minuscule cell populations might be lost during tissue preparation; (2) cannot prove the causal link between the cell populations with similar transcriptomes. Further single cell-based deep somatic lineage tracing will help to address these limitations.

In summary, this study provides direct evidence for a gastric origin for Barrett's esophagus and demonstrated that Barrett's esophagus is a necessary step in the progression of EAC. These findings provide a rationale for the development of early clinical diagnosis and cancer prevention strategies.

## References

[1] Sung H Ferlay J Siegel R L Global Cancer Statistics 2020: GLOBOCAN estimates of incidence and mortality worldwide for 36 cancers in 185 countries CA Cancer J Clin 2021 71 03 209 249 33538338 10.3322/caac.21660

[2] Jiang D Zhang L Liu W Trends in cancer mortality in China from 2004 to 2018: a nationwide longitudinal study Cancer Commun (Lond) 2021 41 10 1024 1036 34251754 10.1002/cac2.12195 PMC8504142

[3] He Y Liang D Du L Clinical characteristics and survival of 5283 esophageal cancer patients: a multicenter study from eighteen hospitals across six regions in China Cancer Commun (Lond) 2020 40 10 531 544 32845581 10.1002/cac2.12087 PMC7571391

[4] Patel N Benipal B Incidence of esophageal cancer in the United States from 2001-2015: a United States cancer statistics analysis of 50 states Cureus 2018 10 12 e3709 30788198 10.7759/cureus.3709 PMC6373890

[5] Qiu H Cao S Xu R Cancer incidence, mortality, and burden in China: a time-trend analysis and comparison with the United States and United Kingdom based on the global epidemiological data released in 2020 Cancer Commun (Lond) 2021 41 10 1037 1048 34288593 10.1002/cac2.12197 PMC8504144

[6] OCCAMS Consortium Sawas T Killcoyne S Iyer P G Identification of prognostic phenotypes of esophageal adenocarcinoma in 2 independent cohorts Gastroenterology 2018 155 06 1720 1.728E7 30165050 10.1053/j.gastro.2018.08.036 PMC6298575

[7] Lu L Mullins C S Schafmayer C Zeißig S Linnebacher M A global assessment of recent trends in gastrointestinal cancer and lifestyle-associated risk factors Cancer Commun (Lond) 2021 41 11 1137 1151 34563100 10.1002/cac2.12220 PMC8626600

[8] Nowicki-Osuch K Zhuang L Jammula S Molecular phenotyping reveals the identity of Barrett's esophagus and its malignant transition Science 2021 373(6556):760 767 34385390 10.1126/science.abd1449

[9] Jiang M Li H Zhang Y Transitional basal cells at the squamous-columnar junction generate Barrett's oesophagus Nature 2017 550(7677):529 533 29019984 10.1038/nature24269 PMC5831195

[10] Wang X Ouyang H Yamamoto Y Residual embryonic cells as precursors of a Barrett's-like metaplasia Cell 2011 145 07 1023 1035 21703447 10.1016/j.cell.2011.05.026 PMC3125107

[11] Leedham S J Preston S L McDonald S A Individual crypt genetic heterogeneity and the origin of metaplastic glandular epithelium in human Barrett's oesophagus Gut 2008 57 08 1041 1048 18305067 10.1136/gut.2007.143339 PMC2564832

[12] Owen R P White M J Severson D T Single cell RNA-seq reveals profound transcriptional similarity between Barrett's oesophagus and oesophageal submucosal glands Nat Commun 2018 9 01 4261 30323168 10.1038/s41467-018-06796-9 PMC6189174

[13] Wang S Zhan M Yin J Transcriptional profiling suggests that Barrett's metaplasia is an early intermediate stage in esophageal adenocarcinogenesis Oncogene 2006 25 23 3346 3356 16449976 10.1038/sj.onc.1209357

[14] Rogerson C Britton E Withey S Hanley N Ang Y S Sharrocks A D Identification of a primitive intestinal transcription factor network shared between esophageal adenocarcinoma and its precancerous precursor state Genome Res 2019 29 05 723 736 30962179 10.1101/gr.243345.118 PMC6499311

[15] Cancer Genome Atlas Research Network Analysis Working Group: Asan University BC Cancer Agency Brigham and Women's Hospital Broad Institute Brown University Case Western Reserve University Dana-Farber Cancer Institute Duke University Greater Poland Cancer Centre Harvard Medical School Institute for Systems Biology KU Leuven Mayo Clinic Memorial Sloan Kettering Cancer Center National Cancer Institute Nationwide Children's Hospital Stanford University University of Alabama University of Michigan University of North Carolina University of Pittsburgh University of Rochester University of Southern California University of Texas MD Anderson Cancer Center University of Washington Van Andel Research Institute Vanderbilt University Washington University Genome Sequencing Center: Broad Institute Washington University in St. Louis Genome Characterization Centers: BC Cancer Agency Broad Institute Harvard Medical School Sidney Kimmel Comprehensive Cancer Center at Johns Hopkins University University of North Carolina University of Southern California Epigenome Center University of Texas MD Anderson Cancer Center Van Andel Research Institute Genome Data Analysis Centers: Broad Institute Brown University Harvard Medical School Institute for Systems Biology Memorial Sloan Kettering Cancer Center University of California Santa Cruz University of Texas MD Anderson Cancer Center Biospecimen Core Resource: International Genomics Consortium Research Institute at Nationwide Children's Hospital Tissue Source Sites: Analytic Biologic Services Asan Medical Center Asterand Bioscience Barretos Cancer Hospital BioreclamationIVT Botkin Municipal Clinic Chonnam National University Medical School Christiana Care Health System Cureline Duke University Emory University Erasmus University Indiana University School of Medicine Institute of Oncology of Moldova International Genomics Consortium Invidumed Israelitisches Krankenhaus Hamburg Keimyung University School of Medicine Memorial Sloan Kettering Cancer Center National Cancer Center Goyang Ontario Tumour Bank Peter MacCallum Cancer Centre Pusan National University Medical School Ribeirão Preto Medical School St. Joseph's Hospital &Medical Center St. Petersburg Academic University Tayside Tissue Bank University of Dundee University of Kansas Medical Center University of Michigan University of North Carolina at Chapel Hill University of Pittsburgh School of Medicine University of Texas MD Anderson Cancer Center Disease Working Group: Duke University Memorial Sloan Kettering Cancer Center National Cancer Institute University of Texas MD Anderson Cancer Center Yonsei University College of Medicine Data Coordination Center: CSRA Inc. Project Team: National Institutes of Health Integrated genomic characterization of oesophageal carcinoma Nature 2017 541(7636):169 175 28052061 10.1038/nature20805 PMC5651175

